# Characterization of genes involved in ceramide metabolism in the Pacific oyster (*Crassostrea gigas*)

**DOI:** 10.1186/1756-0500-5-502

**Published:** 2012-09-13

**Authors:** Emma Timmins-Schiffman, Steven Roberts

**Affiliations:** 1 School of Aquatic and Fishery Sciences, University of Washington, Seattle, WA, 98105, USA

**Keywords:** Oyster, *Crassostrea gigas*, Stress, Ceramide, Bacteria

## Abstract

**Background:**

The lipid signaling molecule, ceramide, is a key component of the vertebrate stress response, however, there is limited information concerning its role in invertebrate species. In order to identify genes involved in ceramide metabolism in bivalve molluscs, Pacific oyster genomic resources were examined for genes associated with ceramide metabolism and signaling.

**Results:**

Several genes were identified including full-length sequences characterized for *serine palmitoyltransferase-1, 3-ketodihydrosphingosine reductase, acid ceramidase, and ceramide glucosyltransferase*. Genes involved in ceramide synthesis and metabolism are conserved across taxa in both form and function. Expression analysis as assessed by quantitative PCR indicated all genes were expressed at high levels in gill tissue. The role of the ceramide pathway genes in the invertebrate stress response was also explored by measuring expression levels in adult oysters exposed to *Vibrio vulnificus*. Two genes demonstrated increased expression during the bacterial challenge: a gene involved in hydrolytic breakdown of ceramide (*acid ceramidase*) and a gene involved in *de novo* generation of ceramide (*3-ketodihydrosphingosine reductase*), suggesting a possible role of ceramide in the invertebrate stress and immune responses.

**Conclusions:**

*In silico* and laboratory results support that Pacific oysters have the basic components of the ceramide metabolism pathway. These results also indicate that ceramide may have analogous functions in vertebrates and invertebrates. The gene expression pattern of *acid ceramidase* and *3-kethodihydrosphingosine reductase* in response to bacterial exposure especially supports that ceramide and sphingolipid metabolism may be involved in the oyster’s stress and/or immune responses.

## Background

Ceramide is a sphingolipid that serves as an important signaling molecule for a variety of cellular processes including differentiation, proliferation, inflammation, and apoptosis [reviewed in 1 and 2]. Different stimuli promote either *de novo* synthesis of ceramide or its catabolic generation from sphingolipids [1,2]. The diversity of processes in which ceramide plays a role as a signaling molecule indicates its importance across a variety of life stages and environmental conditions. For example, the accumulation of ceramide can halt embryonic development [3], inhibit insulin signaling [4], and promote apoptosis during cellular stress [5]. The production of ceramide can be triggered by multiple pathways and is sensitive to exogenous stressors [5,6]. In sea bass (*Diecentrarchus labrax*), changes in intracellular ceramide levels in gill tissue are associated with abrupt shifts in environmental salinity [7]. Leukemia cells exposed to various exogenous stressors (ionizing radiation, hydrogen peroxide, UV radiation, and heat shock) showed elevated levels of ceramide and increased apoptosis [8].

Ceramide metabolism has also been associated with immune-related processes. Cytokines can trigger sphingomyelin hydrolysis, leading to increased production of ceramide, suggesting that ceramide could propagate cytokine signaling [2]. Ceramide also plays a key role in the inflammatory response in *Homo sapiens* dermal fibroblasts by stimulating interleukin-1 mediated prostaglandin E2 production [9].

While the role of ceramide as a signaling molecule in response to stress has been well studied in mammalian systems, there is little information regarding the function and metabolism of ceramide in invertebrates. The primary goal of this study was to characterize genes associated with ceramide metabolism in the intertidal mollusc, the Pacific oyster (*Crassotrea gigas*). Using an *in silico* approach, numerous genes associated with ceramide metabolism were identified and complete coding sequences were isolated for select genes. To provide insight into the functional role of ceramide metabolism in the invertebrate stress response, adult *C. gigas* were exposed to the marine bacterium *Vibrio vulnificus* and the expression levels of four genes involved in the ceramide pathway were assessed. Given the range of environmental conditions experienced by intertidal species, ceramide signaling could be a key component in the cellular response to these environmental changes.

## Results

**Figure 1:**
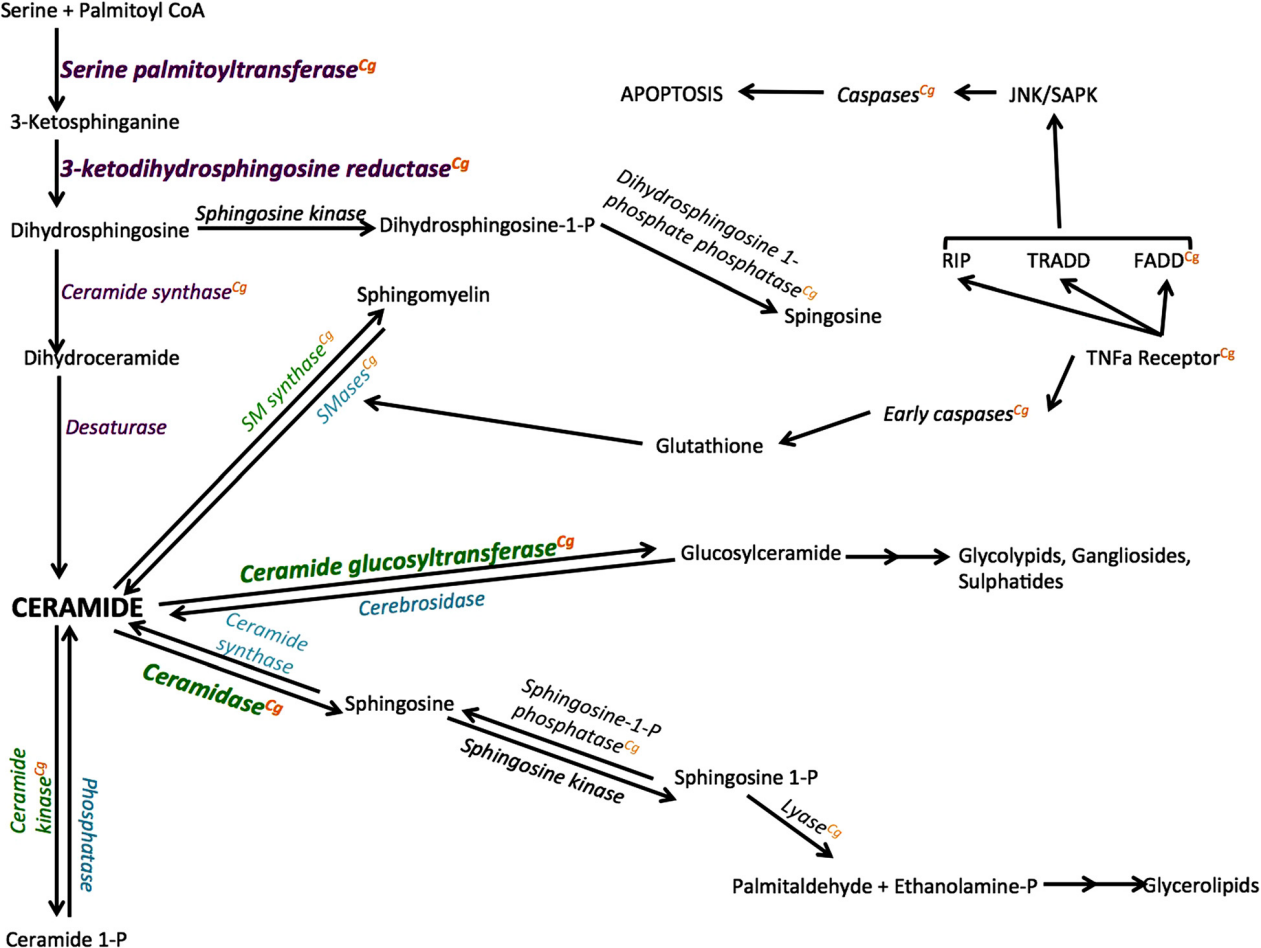
**Representation of the major players in the ceramide metabolism pathways.** Enzymes are in italics with the genes characterized as part of this study in bold: *serine palmitoyltransferase*, *3-ketodihydrosphingosine reductase*, *ceramide glucosyltransferase*, and *acid ceramidase*. Enzymes involved in the *de novo* synthesis of ceramide are in purple. Those implicated in the enzymatic break-down of ceramide are in green and the enzymes responsible for ceramide’s catabolic generation are in blue. Genes that were discovered in the *Crassostrea gigas* transcriptome using bioinformatics have a superscript “Cg” in orange. The pathway is adapted from [2,15].

A total of 23 sequences associated with ceramide meta-bolism were identified by analyzing publicly available *Crassostrea gigas* sequences (Table 1). A majority of the genes are homologous to vertebrate genes involved in *de novo* synthesis, catabolic generation, or enzymatic breakdown of ceramide (Figure 1). Most sequences were derived from contigs generated by assembling short read sequences (see Additional file 1). Of the 23 sequences, 4 were selected for further characterization based on the percent of putative open reading frame identified. These four genes include *serine palmitoyltransferase-1* (*Cg-sptlc1*), *3-ketodihydrosphingosine reductase* (*Cg-3KDSR*), *acid ceramidase* (*Cg-AC*), and *ceramide glucosyltransferase* (*Cg-GlcCer*). Based on amino acid alignments, complete nucleotide open reading frames were obtained for *Cg-sptlc1* [GenBank: JN315146], *Cg-3KDSR* [GenBank: JN315143], and *Cg-AC* [GenBank: JN315144]. *Cg-GlcCer* [GenBank: JN315145] is missing a portion of the 3’ end of the nucleotide sequence as determined from alignments with full-length sequences in other species.

**Table 1:** **Genes associated with ceramide metabolism in *C. gigas *from the *in silico *search **

***Gene*ID**	**Gene description**	***Species***	**e-value**
*Cg_4852*	Serine palmitoyltrasnferase-1	*Pongo abelii*	0
*Cg_877*	Acid ceramidase	*Rattus norvegicus*	2E-146
*Cg_14141*	3-ketodihydrosphingosine reductase	*Mus musculus*	5e-46
*Cg_29918*	Ceramide glucosyltransferase	*Xenopus tropicalis*	9e-122
*Cg_21728*	Acid sphingomyelinase	*Mus musculus*	5e-46
*Cg_16356*	Ceramide kinase	*Homo sapiens*	1e-27
*Cg_16726*	Cerebrosidase	*Mus musculus*	5e-139
*Cg_17230*	Neutral ceramidase	*Oryza sativa*	5e-96
*Cg_1560*	Caspase 7	*Homo sapiens*	4e-10
*Cg_23531*	Caspase 8	*Homo sapiens*	8e-54
*Cg_252*	TNF receptor-associated factor 2	*Mus musculus*	1e-53
*Cg_3248*	TNF receptor-associated factor 3	*Mus musculus*	2e-30
*Cg_31180*	TNF receptor-associated factor 4	*Homo sapiens*	4e-39
*Cg_6808*	Neutral sphingomyelinase	*Caenorhabditis elegans*	4e-10
*Cg_20643*	Dihydrosphingosine 1-phosphate phosphatase	*Schizosaccharomyces pombe*	3e-9
*Cg_26221*	Sphingosine-1-phosphate phosphatase	*Mus musculus*	8e-20
*Cg_7888*	Sphingosine-1-phosphate lyase	*Dictyostelium discoideum*	6e-9
*HS213433*	Sphingomyelin synthase	*Homo sapiens*	1e-100
*HS185280*	Ceramide synthase	*Mus musculus*	5e-83
*HQ425701*	Inhibitor of apoptosis	*Crassostrea gigas*	-
*HQ425703*	Caspase 1	*Crassostrea gigas*	-
*HQ425705*	Caspase 2	*Crassostrea gigas*	-
*HQ425699*	Fas-associated receptor with Death Domain	*Crassostrea gigas*	-

Genes associate with ceramide metabolism were identified in *C. gigas* by searching of publicly available databases. Sequences assembled from short read archive data and expressed sequence tags were given a Gene ID code that corresponds to the sequence in the Additional file 1. For each of these contiguous sequences, the top BLASTx hit description, corresponding species, and e-value are provided. Two genes that matched to a single EST each in GenBank (*sphingomyelin synthase* and *ceramide synthase*) are denoted with their respective GenBank Accession Numbers since they are not represented as a contig of multiple ESTs. An additional four genes [GenBank: HQ425699, HQ425701, HQ425703, and HQ425705] have been previously characterized in *C. gigas*[20].

### Serine palmitoyltransferase-1

**Figure 2:**
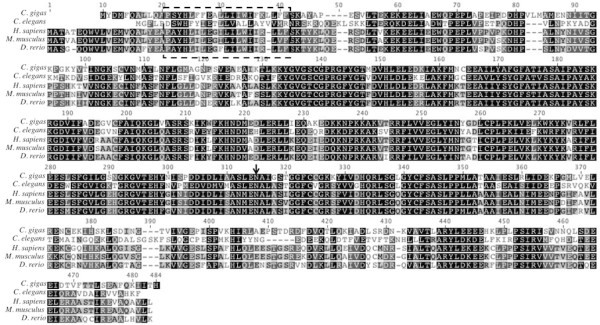
**Amino acid alignment of translated *Cg-sptlc1* with protein sequence of *C. elegans *[GenBank: NP_001021978], *H. sapiens *[GenBank; NP_006406], *M. musculus *[GenBank: NP_033295], and *D. rerio *[GenBank: NP_001018307].** Black shading indicates 100% similarity across sequences, dark gray is 80-100% similarity, light gray is 60-80% similarity, and white is less than 60% similarity. The transmembrane domain is marked by the dashed box and the asparagine that corresponds to the *H. sapiens* LCB1 isoform is marked with an arrow.

The open reading frame of *Cg-sptlc1* is 1404 bp and is most similar to *sptlc-1* in *Xenopus tropicalis* and in the hemichordate, *Saccoglossus kowalevskii* (Table 2). At the amino acid level Cg-sptlc1 is most similar to serine palmitoyltransferase 1 in the Sumatran orangutan, *Pongo abelii* (Table 2). Based on alignments at the deduced amino acid level, Cg-sptlc1 shares 59.8% pairwise identity with the *H. sapiens* homolog and 51.0% pairwise identity over 475 amino acids with *Caenorhabditis elegans* Sptlc1 (Figure 2).

**Table 2:** **Top BLASTn and BLASTx hits for the *Crassostrea gigas *genes *Cg-sptlc1*, *Cg-3KDSR*, *Cg-GlcCer*, *Cg-AC***

**Gene**	**Top BLASTn Hit**	**BLASTn Similarity**	**2**^**nd **^**BLASTn Hit**	**2**^**nd **^**BLASTn Similarity**	**Top BLASTx Hit**
*Cg-sptlc1*	*Xenopus tropicalis (NM_001079574)*	71%	*Saccoglossus kowalevskii (SM_002730516)*	70%	*Pongo abelii (Q5R9T5)*
*Cg-3KDSR*	*Rattus norvegicus (NM_001108342)*	68%	*Saccoglossus kowalevskii (SM_002740331)*	76%	*Mus musculus (Q6GV12)*
*Cg-GlcCer*	*Pediculus humanus corporis (SM_002431306)*	66%	*Xenopus laevis (NM_001090475)*	66%	*Xenopus tropicalis (Q5BL38)*
*Cg-AC*	*Sebastes schlegelii (AB491143)*	67%	-	-	*Rattus norvegicus (Q6P71)*

The BLAST algorithm was used to find the best matches in the NCBI nucleotide database (BLASTn) and UniProt/SwissProt protein database (BLASTx). For each gene, the top two BLASTn matches are given along with the percent sequence similarity. Sequence identity values shown represent local similarity as reported by BLAST. *Cg-AC* only had one top BLASTn hit. The top BLASTx protein hit is also given. GenBank Accession Numbers are in parentheses next to the species name of the top hit.

**Figure 3:**
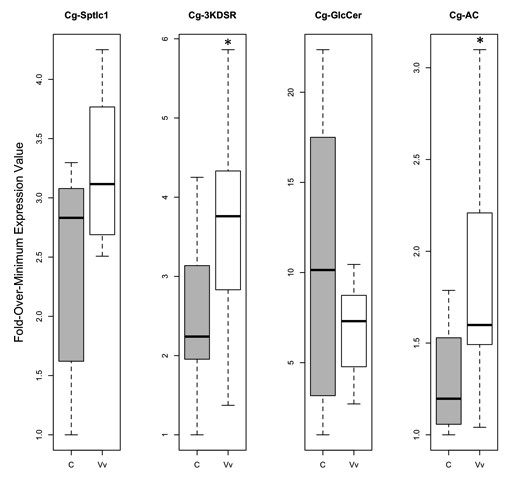
**Expression values in gill tissue for *serine palmitoyltransferase-1*** (***Cg-sptlc1***), ***3-ketodihydrosphingosine reductase *(*Cg-3KDSR*), *ceramide glucosyltransferase *(*Cg-GlcCer*), and *acid ceramidase *(*Cg-AC*).** Gene expression values for the control (“C”, n = 8) oysters are represented by the gray boxes, while the *V. vulnificus*-exposed (“Vv”, n = 8) oysters are represented with the white boxes. Boxes represent the spread of the middle 50% of the data with the median shown as the horizontal black line in the box. The dotted lines span the remaining data. An asterisk indicates a significant difference in expression between exposed and control oysters.

The highest level of *Cg-sptlc1* gene expression was detected in gill tissue, followed by digestive gland, mantle, and then adductor muscle (data not shown). Expression levels in gill tissue were 40 times higher the levels in adductor muscle tissue. *Cg-sptlc1* expression was not significantly altered in gill tissue from oysters exposed to *Vibrio* (mean expression ± standard error = 2.4E-3 ± 2.0E-4), compared to controls (1.8E-3 ± 2.3E-4, p = 0.068; Figure 3). Average gene efficiency calculated in PCR Miner [10] for qPCR was 0.733 (standard deviation = 0.07).

### 3-ketodihydrosphingosine reductase

**Figure 4:**
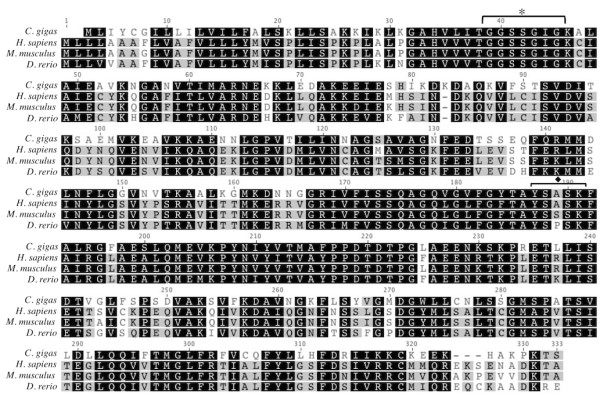
**Amino acid alignment of translated Cg-3KDSR with protein sequence from *H. sapiens *[GenBank: Q06136], *M. musculus *[GenBank: NP_081810], and *D. rerio *[GenBank: NP_957433].** Black shading indicates 100% similarity across sequences, dark gray is 80-100% similarity, light gray is 60-80% similarity, and white is less than 60% similarity. The conserved catalytic site is marked with an asterisk and the NADH/NADPH binding site and active site motif is marked with a diamond.

The *Cg-3KDSR* open reading frame is 1129 bp and is most similar to the *Rattus norvegicus 3KDSR* sequence and to *3KDSR* from *Saccoglossus kowalevskii* (Table 2). The amino acid translation of *Cg-3KDSR* is most similar to *Mus musculus* 3KDSR (Table 2). The *Crassostrea gigas* amino acid sequence shares 50.8% identity to the corresponding homolog in *H. sapiens* (Figure 4). Based on the derived amino acid sequence of Cg-3KDSR, the catalytic site and NADH/NADPH binding site [11] are conserved in oysters (Figure 4).

Gene expression of *Cg-3KDSR* was highest in gill tissue with expression levels over 1000 times higher compared to other tissues (data not shown), which had comparable expression of this gene. *Cg-3KDSR* gene expression in *Vibrio*-exposed oysters (8.4E-5 ± 1.1E-5) was higher than expression in controls (5.8E-5 ± 8.5E-6, p = 0.037; Figure 3). Average gene efficiency calculated in PCR Miner [10] was 0.918 (standard deviation = 0.08).

### Ceramide glucosyltransferase

**Figure 5:**
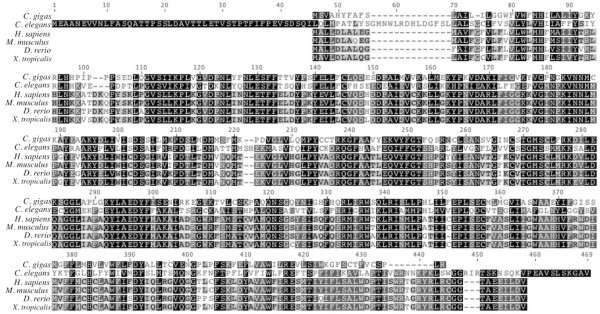
**Amino acid alignment of translated Cg-GlcCer with ceramide glucosyltransferase protein sequences from *C. elegans *[GenBank: NP_506971], *H. sapiens *[GenBank: NP_003349], *M. musculus *[GenBank: NP_035803], and *X. tropicalis* [GenBank: Q5BL38].** Black shading indicates 100% similarity across sequences, dark gray is 80-100% similarity, light gray is 60-80% similarity, and white is less than 60% similarity.

*Cg-GlcCer* (1124 bp) is most similar to *ceramide glucosyltransferase* from the human body louse, *Pediculus humanuscorporis*, followed by *Xenopus laevis UDP-glucose ceramide glucosyltransferase* (Table 2). The translated amino acid sequence is most similar to *Xenopustropicalis* ceramide glucosyltransferase (Table 2). *Crassostrea gigas* and *H. sapiens* share a 45.9% pairwise amino acid identity over 396 residues in the alignment, while *C. elegans* and *C. gigas* share 40.9% pairwise identity over 468 residues (Figure 5).

*Cg-GlcCer* had a similar expression profile across tissues to *Cg-sptlc1*, the highest expression being in the gill, followed by digestive gland, mantle and adductor (data not shown). The gene expression data for the bacterial exposure were square-root transformed based on a λ of 0.42. The *Cg-GlcCer* gene was not expressed differently in *Vibrio*-exposed oysters (2.4E-8 ± 3.3E-9) compared to control oysters (3.8E-8 ± 1.1E-8, p = 0.98; Figure 3). Average gene efficiency calculated in PCR Miner [10] was 0.898 (standard deviation = 0.16).

### Acid ceramidase

**Figure 6:**
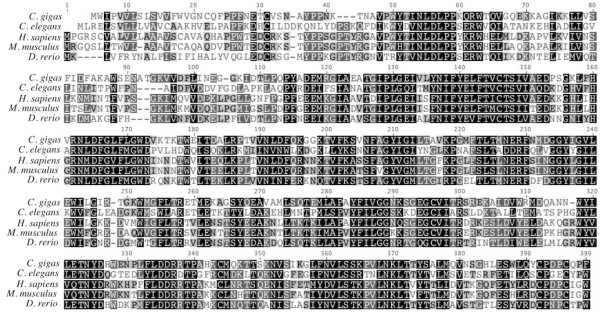
**Amino acid alignment of translated Cg-AC with protein sequences from acid ceramidase in *C. elegans *[GenBank: NP_493173], *H. sapiens *[GenBank: NP_808592], *M. musculus [GenBank: NP_062708], and D. rerio* [GenBank: NP_001006088].** Black shading indicates 100% similarity across sequences, dark gray is 80-100% similarity, light gray is 60-80% similarity, and white is less than 60% similarity.

The open reading frame for *Cg-AC* is 1170 bp in length and was most similar to the gene *BRF 7-G7* in *Sebastes schlegelii* (Table 2). The translated amino acid sequence for *C. gigas* is the most similar to *R. norvegicus* acid ceramidase (Table 2). The Cg-AC amino acid sequence shares 46.6% pairwise identity over 402 residues in the alignment with *C. elegans* and 49.4% identity with the *H. sapiens* sequence over 398 residues (Figure 6).

*Cg-AC* was expressed the most in the gill tissue followed by digestive gland, mantle, and adductor (data not shown). The gene expression data for the bacterial exposure were subjected to a reciprocal transformation based on a λ of −0.87. The expression of *Cg-AC* was significantly higher in *Vibrio*-exposed oysters (2.2E-3 ± 2.8E-3) compared to controls (1.6E-3 ± 1.3E-4, p = 0.038; Figure 3). Average gene efficiency calculated in PCR Miner [10] was 0.876 (standard deviation = 0.06).

**Figure 7:**
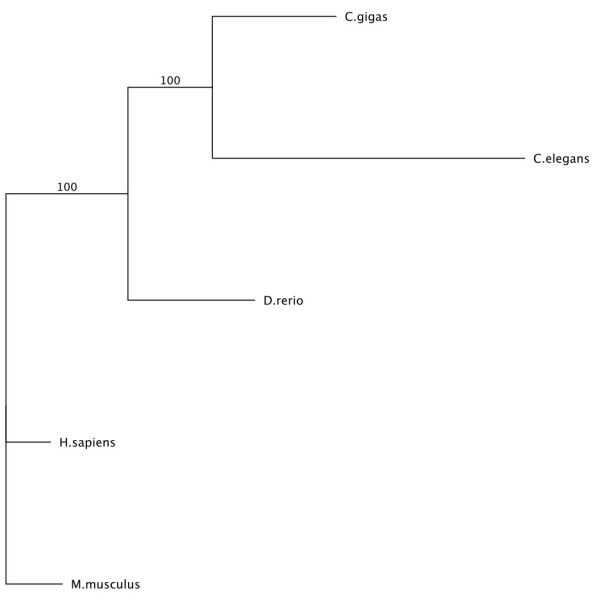
**Maximum likelihood phylogenetic tree of the amino acid alignment of acid ceramidase in *C. gigas***, ***C. elegans, H. sapiens, M. musculus*, and *D. rerio.*** All other protein trees had similar topology to the one shown. The tree was created based on the James-Taylor-Thornton (JTT) model and bootstrapped 100 times.

All four genes showed similar phylogenetic topologies (Figure 7), with the amino acid sequences clustering into distinct invertebrate and vertebrate lineages. When the *C. elegans* sequence was available and included in the phylogeny, it clustered with the *C. gigas* sequence with a bootstrap value of 100%. *H. sapiens* and *M. musculus* sequences always clustered together with a bootstrap of 100%.

## Discussion

### Gene identification

This study identified a suite of genes in the Pacific oyster homologous to vertebrate genes associated with ceramide metabolism, including the sequencing and characterization of *serine palmitoyltransferase-1* (*Cg-sptlc1*), *acid ceramidase* (*Cg-AC*), *3-ketodihydrosphingosine reductase* (*Cg-3KDSR*), and *ceramide glucosyltransferase* (*Cg-GlcCer*). These data provide an important resource for further studies that focus on the role of ceramide in the environmental response in invertebrates. While well studied in vertebrate systems, there have been only a few recent studies that focus on ceramide metabolism and signaling in molluscs [see [12-14].

Numerous genes associated with ceramide metabolism are conserved across distant taxonomic lineages. In vertebrates, the genes described here are directly responsible for synthesis of ceramide (sptlc1, 3KDSR; Figure 1) and generation of sphingolipids from ceramide (AC and GlcCer). *In silico* analysis of the *Crassostrea gigas* transcriptome shows that there are a number of other genes in these ceramide metabolism pathways (Table 1). In fact, almost all the genes coding for enzymes necessary for *de novo* ceramide synthesis were identified, suggesting a conservation of this metabolic pathway in *C. gigas*. The only gene not found was that coding for the enzyme desaturase that converts dihydroceramide to ceramide (Figure 1). However, further research into the presence, structure, and functional role of ceramide is needed to confirm these suggestive results. Additionally, a number of enzymes responsible for transformation of ceramide into other lipid products were identified including ceramide kinase, ceramide synthase , and sphingomyelin synthase. A variety of caspases, TNF superfamily receptors, RIP (receptor-interacting serine/threonine-protein) and FADD (Fas-associated protein with death domain) subunits of the TNFα (tumor necrosis factor) receptor, which are key components of the cellular stress and apoptotic responses, were also identified in public databases. These receptors are implicated up- and downstream of ceramide signaling pathways in vertebrates [2,15]. Several genes known to be involved in ceramide metabolism were not found in this effort (i.e. dihydroceramide desaturase, ceramide-1P phosphatase). This is likely related to the fact that these genes have yet to be sequenced in the Pacific oyster. It is also possible that corresponding enzymes lack significant sequence homology. Once a complete genome is sequenced for this species, a more comprehensive analysis can be performed.

### Gene characterization

Sptlc1, responsible for accumulation of intracellular ceramide during cellular stress [5,16], is highly conserved in oysters including a 21 residue transmembrane region originally identified in *H. sapiens*[17]. The serine palmitoyltransferase identified in *C. gigas* has high homology with the LCB1 *H. sapiens* isoform. There are two forms of *H. sapiens* Sptlc – LCB1 and LCB2 [17]. *Homo sapiens* LCB2 has a conserved motif that binds pyridoxal phosphate [17,18], but LCB1 has an asparagine instead, which is homologous to the *C. gigas* sequence. In *Homo sapiens*, LCB1 is necessary for the maintenance of LCB2 and does not perform the same catalytic functions [17]. More research is needed to determine if the functionality of specific Sptlc isoforms are conserved across taxa.

*Crassostrea gigas* 3KDSR shares conserved catalytic domains with all other amino acid sequences in the alignment, suggesting that its functionality is conserved across taxonomic groups. 3-ketodihydrosphingosine reductase acts downstream of Sptlc. The product of the reaction catalyzed by Sptlc is 3-ketosphinganine. 3-ketosphinganine is reduced by a NADPH-dependent reductase to dihydrosphingosine. The enzyme that catalyzes this reaction is 3-ketodihydrosphingonsine reductase (3KDSR). 3KDSR contains two functional sites that are highly conserved, based on the amino acid alignment: an NADH/NADPH binding site and a catalytic site [11] (Figure 4). All four amino acid sequences – *C. gigas, H. sapiens, M. musculus,* and *D. rerio* - share more of the catalytic site motif than previously described: Tyr-Ser-X-Ser-Lys, beginning at position 187 on the alignment [11] (Figure 4). The motif of the NADH/NADPH binding site is identical in its entirety – Gly-Gly-Ser-Ser-Gly-Ile-Gly – across all four sequences/taxa [11] (Figure 4).

### Implications for the stress response

The gene expression patterns observed for each gene suggest a role of ceramide metabolism in the oyster stress response. The highest expression was observed in the gill tissue, which is rich with hemocytes, the primary immune cell in bivalves. Furthermore, exposure to *Vibrio vulnificus* significantly elevated expression of *Cg-AC* and *Cg-3KDSR*. There are several possible interpretations of this increase in gene expression that corroborate the second messenger role of ceramide in oysters. The increased expression of both enzymes suggests that the bacterial exposure prompted up-regulation of ceramide production (effected by *Cg-3KDSR*) followed by its metabolism into other sphingolipids (by *Cg-AC*). There are a number of reasons why ceramide may be transformed into other lipid products during environmental stress. For instance, increased expression of *Cg-AC* could indicate that ceramide is transformed into the lipid sphingosine, which then functions downstream as a signaling molecule in the *C. gigas* immune response. Sphingosine is an important signaling molecule in the vertebrate immune response and probably plays a similar role in invertebrates. Sphingosine is associated with the inhibition of the proliferation of Th2 T cells, inhibition of protein kinase C activity, regulation of the complement system, and inhibition of neutrophil respiratory burst [19,20]. Bivalves possess a complex immune system that has many homologous components to the vertebrate immune system [21], thus it is probable that secondary signaling molecules like ceramide are instrumental in immune response coordination. The roles that sphingosine and other sphingolipids play in the immune response seem to be heavily influenced by their concentrations [20], thus *Cg-AC* could be a pivotal enzyme regulating levels of sphingosine in oyster, at least in a short-term response.

An alternative explanation for the increased expression of *Cg-AC* during *Vibrio* vulnifcus challenge suggests that ceramide is the primary signaling molecule in the *C. gigas* immune response. An accumulation of ceramide in response to the *V. vulnificus* exposure could have occurred and *Cg-AC* may be up-regulated to metabolize ceramide after it has performed its signaling roles. Ceramide may have been produced to increase signaling of immune pathways necessary for responding to bacterial exposure. Increased expression of AC has been shown to decrease intracellular ceramide in mammals [4,6] and could very well play the same role in invertebrates. The relatively greater expression of *Cg-3KDSR* in *Vibrio*-exposed oysters supports this second hypothesis.

## Conclusions

Here we report the identification of numerous genes in *Crassostrea gigas* that are homologous to genes involved in vertebrate metabolism of ceramide, an important lipid signaling molecule. Gene expression analysis suggests that ceramide is involved in the immune response of oysters exposed to microbial pathogens. It should be noted that a limited number of genes were examined here and targeted studies would be required to further elucidate the functional role of ceramide metabolism in bivalves. For instance, future efforts might directly quantify sphingolipid levels and correlate levels with specific cellular function or use of fluorescence *in situ* hybridization to show cellular gene expression. Assays of gene expression are sometimes merely suggestive of a true, functional organismal response effected by changes in protein expression. Changes in global gene expression, however, are indicative of a significant physiological response, at least at the cellular level, and frequently these changes are correlated with changes in protein expression [22]. It is not known if lipid content in bivalve diets impacts stress physiology by influencing ceramide levels. Le Grand *et al*. [23] discovered that ceramide-based phosphophingolipids are an important component of Pacific oyster hemocyte membranes. Characterizing how diet and other conditions affect ceramide metabolism could offer a framework for better understanding mechanisms associated with environmental effects on immune function.

## Methods

### Gene discovery

Genes involved in *Crassostrea gigas* ceramide metabolism were identified using publicly available sequence data. Specifically, short read sequences from *C. gigas* larvae complementary DNA (cDNA) libraries [GenBank: SRX032364] as well as all expressed sequence tags (ESTs) were downloaded from NCBI ( http://www.ncbi.nlm.nih.gov). All sequences were quality trimmed and *de novo* assembled using CLC Genomics Workbench v3.7 (CLC bio, Katrinebjerg, Denmark). Consensus sequences from short read and EST assemblies were compared to the UniProtKB/Swiss-Prot database ( http://www.uniprot.org) using NCBI’s BLASTx algorithm [24]. Sequences having a top blast hit with an e-value less that 1E-5 were inspected for genes associated with ceramide metabolism. Only genes with an e-value less than 1E-30 were considered for sequencing and gene expression characterization in this study. Individual sequence alignments were performed to determine percent coverage and sequence similarity (Geneious Pro v. 4.8.5 [25]). Four genes were chosen for sequencing and quantitative PCR (qPCR) analysis: *serine palmitoyltransferase-1*, *3-ketodihydrosphingosine reductase*, *acid ceramidase*, and *ceramide glucosyltransferase*.

### Bacterial challenges

Adult *C. gigas* (mean length = 9.0 cm, range of 7.9-10.3 cm with standard deviation of 0.58 cm) were obtained from Taylor Shellfish Farms, Inc. (Quilcene, WA) and acclimated to lab conditions for a few weeks. For bacterial challenges, *Vibrio vulnificus* was grown in 400 mL culture medium (1x standard Luria-Bertani broth with an additional 1% NaCl) at 37°C for 18 hours at 150 rpm. The culture was then centrifuged for 10 minutes at 4300 rpm (25°C), the supernatant was removed and the pelleted bacteria were resuspended in 50 mL non-sterile of seawater. Eight oysters held in 8 L of aerated seawater were inoculated with *V. vulnificus* at an initial concentration of 4.56x10^18^ CFU/L via a 3 hour immersion bath. Control oysters (n = 8) were likewise placed in 8 L of aerated seawater. Following exposure, oysters were harvested aseptically and gill tissue was dissected and immediately frozen at −80°C. Tissues from a single, non-exposed oyster (gill, mantle, adductor muscle, and digestive gland) were dissected using sterile techniques and stored in RNAlater (Ambion, Carlsbad, CA) for use in a qPCR assay of differential gene expression across tissues. RNA isolation, reverse transcription and quantitative PCR analysis were carried out as described below.

### Gene sequencing

RNA isolation was carried out using Tri-Reagent (Molecular Research Center, Cincinnati, OH) per the manufacturer’s protocol. Following RNA isolation, samples were treated with the Turbo DNA-free Kit, rigorous protocol (Ambion) to remove potential genomic DNA carry-over. All samples were evaluated to ensure genomic DNA was absent by performing quantitative polymerase chain reaction (qPCR) on DNAsed RNA samples. Quality and quantity of RNA were determined using a NanoDrop ND-1000 Spectrophotometer (Thermo Fisher Scientific, Waltham, MA). Equal quantities of DNased RNA samples were reverse transcribed using M-MLV reverse transcriptase according to manufacturer’s protocol (Promega, Madison, WI).

For genes where the putative open reading frame could be determined based on sequence alignments, PCR primers were designed to amplify entire coding regions (Primer 3 in Geneious Pro v. 4.8.5; [25,26]) (Table 3). Equal quantities of within-treatment gill cDNA were pooled for sequencing PCR reactions. PCR reactions (25 μl) were carried out with 12.5 μL 2x Apex RED Taq Master Mix (Genesee Scientific, San Diego, CA), 8.5 μL nuclease-free water, 0.5 μL of 10 μM forward and reverse primers (Integrated DNA Technologies, Coralville, IA), and 3 μL cDNA template. Thermal cycling parameters were as follows: 95°C for 10 minutes; 40 cycles of: 95°C for 30 seconds, 55°C for 30 seconds, and 72°C for 30 seconds; 72°C for 10 minutes. PCR products were separated on agarose gels, checked for expected amplicon size, excised, cloned in pCR 2.1-TOPO Vector, and transformed in to One Shot Top10 chemically competent cells using the TOPO TA Cloning Kit (Invitrogen, San Diego, CA). Plasmid DNA was isolated from bacterial cultures using the Qiagen MiniPrep Kit, following the manufacturer’s protocol (Qiagen, Valencia, CA) and sequenced at the High Throughput Genomics Unit (University of Washington) on an Applied Biosystem’s 3730xl (Carlsbad, CA) using vector-specific primers (Invitrogen).

**Table 3:** Sequencing and qPCR primers for ceramide metabolism genes

**Gene description**	**Elongation factor 1 α (EF1α)**	**Serine palmitoyltransferase (Sptlc1)**	**3-ketodihydrosphingosine reductase (3KDSR)**	**Acid ceramidase (AC)**	**Glucosylceramide synthase (GlcCer)**
Sequencing Forward Primer	-	ATGGCGTCGACGTTCA TTCC	AGCGACGGACCGAACTTACT	TGTGATTACACGAT GTGGATACCG	AGAGGCGAACACA CGAAAGT
Sequencing Reverse Primer	-	CTGTTCCCCAATATTTC TGAC	TGTCTTGGGTTTTGCATCCTTC	CTGCTTCTGACTTC CGGTGT	CCATATGGATAACAC TTCTCG
Sequence Product Size (bp)	-	1483	1210	1165	1080
qPCR Forward Primer	AAGGAAGCTGCTGA GATGGG	TTCACAGCAAGCT GAGCGAT	GCAGTGCAGTGGCTGGAAAT	TGGACTCAAGTTGG CCAGGA	TTGGCCCAAACGGG AAAGTT
qPCR Reverse Primer	CAGCACAGTCAG CCTGTGAAGT	AAGTAGCGAGCC AACGTCAC	AGGCAGCCTTGGTGACATTG	AAGGCTGGGGGAG AGTATCG	TGTCCATGAGCGAGT CTGGT
qPCR Product Size (bp)	200	178	168	157	114

### Protein phylogeny

Sequences were trimmed to their open reading frame and translated to their amino acid sequences (Geneious Pro v. 4.8.5 [25]). Sequence alignments were performed using ClustalX v. 2.1 [27]. Sequences for corresponding proteins in *Homo sapiens, Mus musculus, Danio rerio, Xenopus tropicalis* and *Caenorhabditis elegans* were downloaded from NCBI’s Homologene database where available. Using the PhyML plugin in Geneious [25,28], maximum likelihood phylogenetic trees of the protein sequences were constructed based on the James-Taylor-Thornton (JTT) model and bootstrapped 100 times [28,29].

### Quantitative PCR

Expression levels across tissue types (n = 1) and in response to bacterial exposure (n = 8 per treatment) of all four genes and of the housekeeping gene, *elongation factor 1*α (*EF1α*), were quantified using qPCR. DNA-free RNA was reverse transcribed to cDNA as described above. qPCR was performed using 1μL of cDNA diluted 1:20 in nuclease-free water in a 25μL reaction containing 12.5 μL of 2x Immomix Master Mix (Bioline, London, UK), 0.5 μL of 10 μM forward and reverse primers, 1.0 μL 50 μM SYTO13 (Invitrogen), and 9.5 μL nuclease-free water. Primers used for qPCR are listed in Table 3. Thermal cycling and fluorescence detection were performed using a CFX96 Real-Time Detection System (Bio-Rad, Hercules, CA). Cycling parameters were as follows: 95°C for 10 minutes; 40 cycles of 95°C for 15 seconds, 55°C for 15 seconds, 72°C for 30 seconds. Immediately after cycling, a melting curve protocol was run to verify that a single product was generated in each reaction. Duplicate runs for each gene were always run on the same plate to avoid inter-run variability.

Average Ct (fluorescence-based cycle threshold) values across replicates and average gene efficiencies were calculated with PCR Miner ( http://www.miner.ewindup.info/version2[10]). Gene expression (R_0_) was calculated based on the equation R_0_ = 1/(1 + E)^Ct^, where E is the average gene efficiency and Ct is the cycle threshold for fluorescence. Each primer pair amplified a single product, as demonstrated by a single melting curve (see Additional file 2). All expression values were normalized to expression of *EF1α* [GenBank: AB122066]. *EF1α* did not show differential expression between treatments as verified by a *t*-test done on expression values of the qPCR run in duplicate. All qPCRs were run in duplicate and significant differences in expression were determined using a linear model with α = 0.05. Box-Cox plots were used to assess skewness of gene expression data and determine if transformations needed to be made. The need to transform the data and the transformation used were determined from the lamda (λ) corresponding to the maximum log likelihood in the Box-Cox plot. If λ = 1 fell within the 95% confidence interval of the maximum log likelihood, then no transformation was used. All statistical analyses were done in R [30].

## Availability of supporting data

The data set supporting the results of this article is included within the article (and its additional files).

## Competing interests

The authors declare that they have no competing interests.

## Authors’ contributions

ETS participated in the design of the study, carried out all of the molecular lab work and data analysis, and helped to draft the manuscript. SR helped in the design of the study in and in drafting and revising the manuscript. Both authors read and approved the final manuscript.

## Supplementary Material

Additional file 1 **Contigs of ESTs corresponding to ceramide metabolism pathway genes identified in *C. gigas.*** Contigs are listed by Gene ID and described in Table 1. Click here for file

Additional file 2 **Melting curves for qPCR done on the 4 genes.** Panel A is the melting curve for *Cg-3KDSR*, panel B is for *Cg-AC*, panel C is for *Cg-GlcCer*, and panel D is for *Cg-sptlc1.* Click here for file
