# Supplementary figures and images for: Characterization of genes involved in ceramide metabolism in the Pacific oyster (Crassostrea gigas)
Source: BMC Res Notes. 2012 Sep 13;5:502. doi: 10.1186/1756-0500-5-502 (PMC3517309; doi:10.1186/1756-0500-5-502)

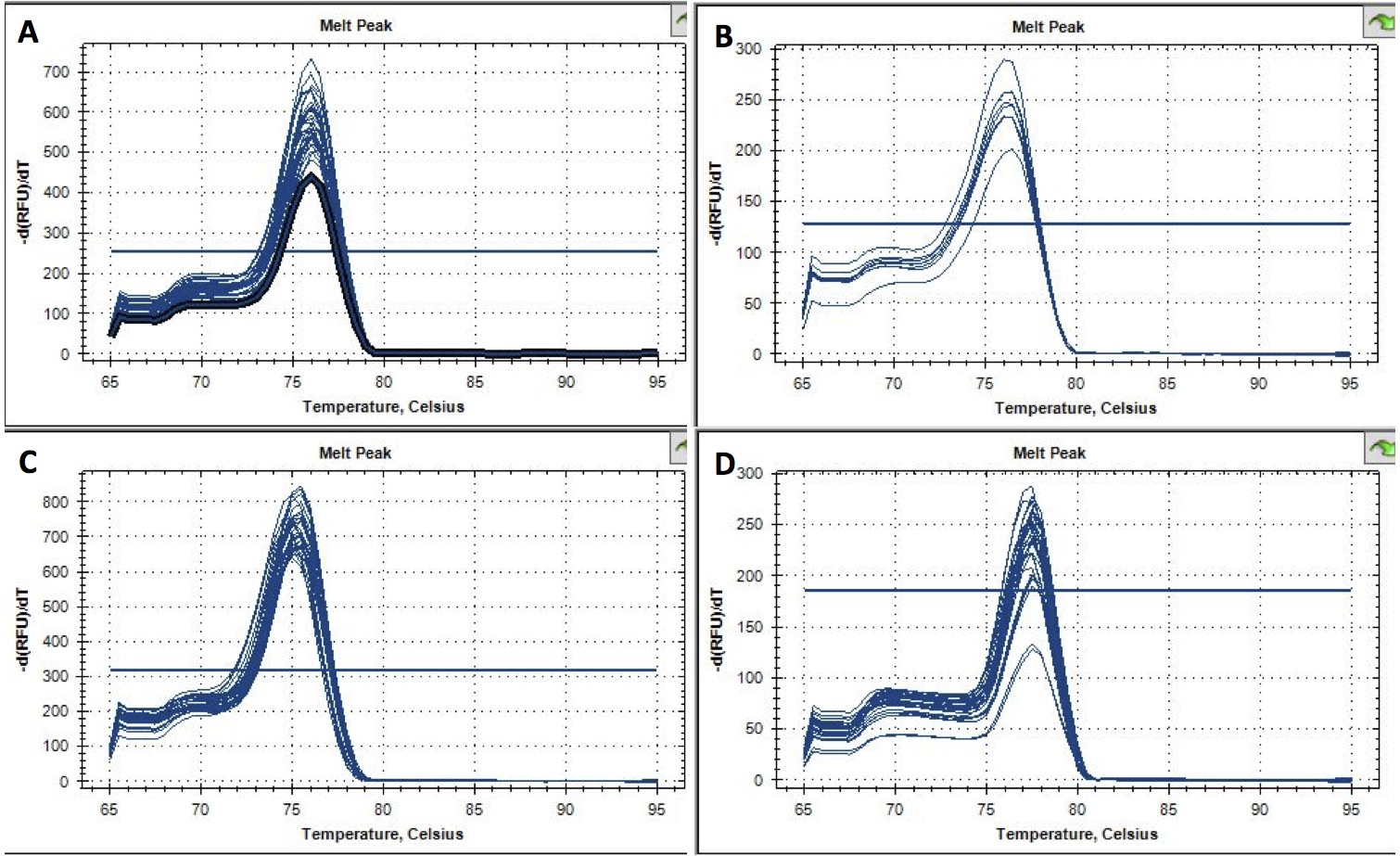

Supplement: Additional file 2 — Melting curves for qPCR done on the 4 genes. Panel A is the melting curve for Cg-3KDSR, panel B is for Cg-AC, panel C is for Cg-GlcCer, and panel D is for Cg-sptlc1. [file 1756-0500-5-502-S2.jpeg]
